# Mesenteric lymph nodes as alternative site for pancreatic islet transplantation in a diabetic rat model

**DOI:** 10.1186/s12893-018-0452-x

**Published:** 2019-04-24

**Authors:** Massimiliano Veroux, Rita Bottino, Roberta Santini, Suzanne Bertera, Daniela Corona, Domenico Zerbo, Giovanni Li Volti, Burcin Ekser, Lidia Puzzo, Marco Raffaele, Salvatore Lo Bianco, Alessia Giaquinta, Pierfrancesco Veroux, Luca Vanella

**Affiliations:** 1 Vascular Surgery and Organ Transplant Unit, Department of Medical and Surgical Sciences, University Hospital of Catania, Via Santa Sofia, 84 95123 Catania, Italy; 2 Institute of Cellular Therapeutics, Allegheny-Singer Research Institute, Allegheny Health Network, Pittsburgh, PA USA; 3 Department of Biomedical and Biotechnological Sciences, Section of Medical Biochemistry, University of Catania, Catania, Italy; 4 Transplant Division, Department of Surgery, Indiana University School of Medicine, Indianapolis, USA; 5 Section of Anatomic Pathology, Department od Medical and Surgical Sciences, and Advanced Technologies, University Hospital of Catania, Catania, Italy; 6 Department of Drug Science, Biochemistry Section, University of Catania, Catania, Italy; 7 Unit of Endocrine Surgery, University Hospital of Catania, Catania, Italy

**Keywords:** Type 1 diabetes mellitus, Islet, Transplantation, Lymph nodes, Mesenteric, Pancreas, Gastric sub-mucosal space, Portal vein, Glycemia

## Abstract

**Background:**

Islet transplantation has progressively become a safe alternative to pancreas transplantation for the treatment of type 1 diabetes. However, the long-term results of islet transplantation could be significantly increased by improving the quality of the islet isolation technique even exploring alternative islet transplantation sites to reduce the number of islets required to mitigate hyperglycemia. The goal of the study was to test the lymph node as a suitable anatomical location for islet engraftment in a rodent model.

**Methods:**

Forty Lewis rats, 6–8 weeks old, body weight 250–300 g, have been used as islet donors and recipients in syngeneic islet transplantation experiments. Ten rats were rendered diabetic by one injection of 65 mg/Kg of streptozotocin. After pancreas retrieval from non diabetic donors, islet were isolated and transplanted in the mesenteric lymph nodes of 7 diabetic rats. Rats were followed for 30 days after islet transplantation.

**Results:**

A total of 7 islet transplantations in mesenteric lymph nodes have been performed. Two rats died 24 and 36 h after transplantation due to complications. No transplanted rat acquired normal glucose blood levels and insulin independence after the transplantation. However, the mean blood levels of glycemia were significantly lower in transplanted rats compared with diabetic rats (470.4 mg/dl vs 605 mg/dl, p 0.04). Interestingly, transplanted rats have a significant weight increase after transplantation compared to diabetic rats (mean value 295 g in transplanted rats vs 245 g in diabetic rats, *p* < 0.05), with an overall improvement of social activities and health. Immunohistochemical analysis of the 5 mesenteric lymph nodes of transplanted rats demonstrated the presence of living islets in one lymph node.

**Conclusions:**

Although islet engraftment in lymph nodes is possible, islet transplantation in lymph nodes in rats resulted in few improvements of glucose parameters.

## Background

Xenotransplantation has recently emerged as a promising alternative to contrast the increasing demand for organ transplantation, and this resulted in many potential clinical applications [1], including the treatment of type 1 diabetes. The dramatic impact on the health of the world’s population has stimulated the need to develop alternative strategies for the treatment of the type 1 diabetes [2]. Pancreatic islet transplantation is becoming an increasingly important strategy for type 1 diabetes mellitus treatment, with a growing number of new procedures performed worldwide, and is able to normalize blood glucose or HbA1c levels in most recipients with diabetes [3–9]. Transplantation of deceased human donor islets into the portal vein in patients with type 1 diabetes mellitus has had encouraging results [3–11], but is associated with an immediate loss of 60–80% of islets through an inflammatory response known as the instant blood-mediated inflammatory reaction (IBMIR) [12, 13]. The long-term results of islet transplantation, therefore, could be significantly increased by improving the quality of the islet isolation technique to increase the yield from the donor pancreas, or reducing the number of islets required to improve hyperglycemia. In recent years, many studies explored alternative islet transplantation sites to reduce the number of islets required to mitigate hyperglycemia [14–18].

The gastric sub-mucosal space (GSMS) is a promising alternative site for islet implantation [14–16]: islets are transplanted through an endoscopic placement, and they would benefit from a rich blood supply and a high oxygen tension. Preclinical trials using a porcine model showed reduced early islet loss and insulin requirements when GSMS islets were compared to intraportal islets [14, 15], and islets were easily accessible for endoscopic monitoring of graft rejection [16].

As alternative sites to the liver, Komori et al [19] demonstrated the surprising potential of lymph nodes (LN) as transplant sites for multiple tissues, among which they also tested pancreatic islets. The goal of this study was to conduct a pilot experiment of feasibility of islet transplantation in the rat model. Following the results of the study by Komori et al [19], where LN was successfully used for islet engraftment in a mouse model, we tested, as a proof of concept, that successful engraftment could be achieved in a larger rodent model.

## Methods

### Experimental procedures

Forty Lewis male rats (Charles River Laboratories, Calco, Italy), 6–8 weeks old, body weight 250–300 g, have been used as islet donors and recipients in syngeneic islet transplantation experiments. From previous publications [20, 21], we estimated that at least 3 pancreata were needed for a single islet transplantation and, consequently, rats were randomized on a 3:1 basis to serve as donor or recipients of a islet transplantation. Ten rats were rendered diabetic by one injection (i.v.) of 65 mg/Kg of streptozotocin (STZ), following the procedure described by Bottino et al. [20]. Blood glucose was measured using a commercial glucometer. Three consecutive blood glucose levels > 350 mg/dl following STZ administration confirmed the diabetic status. Being all syngeneic transplantations, recipients did not receive immunosuppressive therapy.

The goal of the study was to test the lymph node as a suitable anatomical location for islet engraftment. The syngeneic transplantation model was chosen to determine whether the metabolic performance of islets was sufficient to normalize hyperglycemia following transplantation, warranting islet survival and function. Each recipient received islets from 2 or 3 donors, with a total of about 600–900 islets/recipient, depending on the number and the quality of retrieved islets. All rats were housed in microisolator caging in a specific pathogen free environment under the guidelines of the Italian National Institute of Health for animal care. All rats were followed with daily measurement of body weight, water intake and blood glycaemia. All surviving rats were euthanized 30 days after transplantation, by the means of CO_2_ inhalation at a flow rate set to 20% of the chamber volume per minute.

#### Isolation and transplantation reagents

The isolation and transplantation reagents used for experimental procedures have been previously described in details [21]. In brief, tissue culturing media consists of RPMI 1640 media supplemented with 20 mM Hepes, 1% L-glutamine, 1% penicillin/streptomycin, 50 μM b-mercaptoethanol (2-ME) and 10% heat-inactivated fetal bovine serum (FBS, Gibco®). Whole islets were maintained in complete medium with a density of ~ 150 islets in 5 ml medium at 37 °C in an atmosphere of 95% air and 5% CO_2_. Media was replaced every 2–3 days. Due to variability in the activity of different lots of collagenase, all collagenase used in this study (Collagenase type V) was from a single lot. Hanks’ Balanced Salt Solution with calcium and magnesium was supplemented with either 20 mM Hepes (HBSS-Hepes) or 20 mM Hepes plus 0.2% BSA (HBSS-BSA) then filter sterilized. Ficoll was dissolved at the rate of 33.35 g Ficoll per 100 ml HBSS-Hepes. All gradients are mixed thoroughly and stored at 4 °C.

#### Organ procurement

**Fig. 1:**
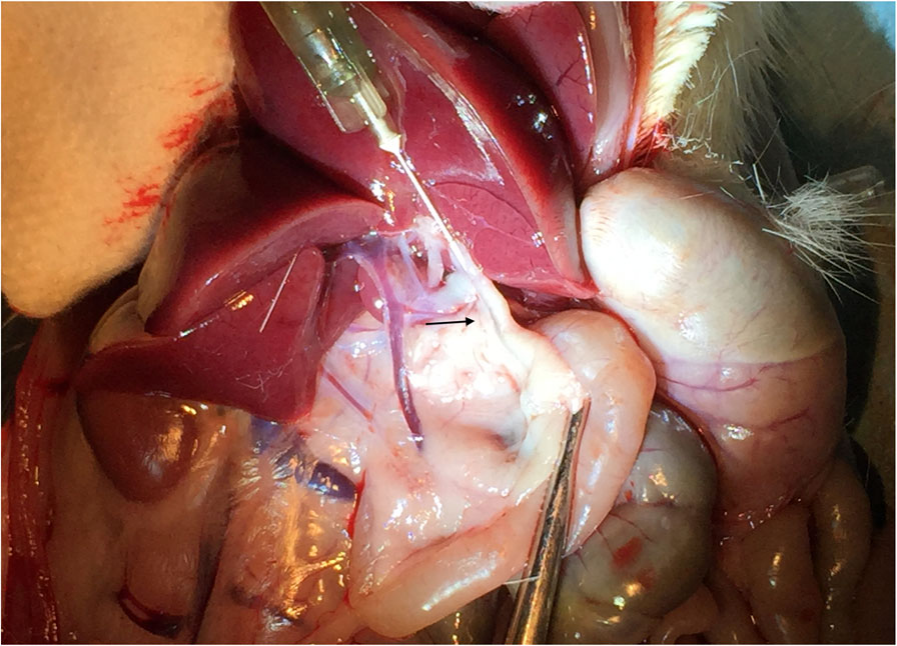
The needle is inserted into the bile duct (arrow)

**Fig. 2:**
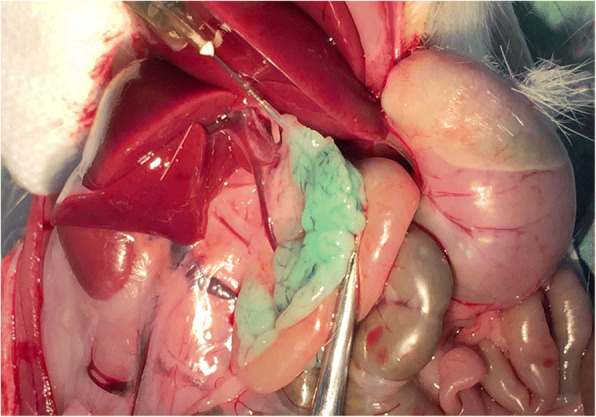
The collagenase is injected into the pancreas until it is fully and visibly distended. A green vegetable dye is added to the collagenase for visibility

Donor rat was sacrificed immediately before pancreas is procured through CO_2_ inhalation at a flow rate set to 20% of the chamber volume per minute. The procurement and isolation procedure was carried out under sterile conditions in a biological safety cabinet. A laparotomy is performed and the skin and body wall are pulled back toward the head. The liver is reflected back against the diaphragm. The common bile duct is located and tied off at the papilla of Vater to prohibit flow from the bile duct into the intestine. A 10 ml syringe is filled with cold collagenase solution and a 25G needle is attached. The needle is inserted into the bile duct, close to the liver with the needle pointed toward the intestine (Fig. 1). The needle is inserted into the bile duct as far as possible without further damaging the duct (no other punctures). The collagenase is injected into the pancreas until it is fully and visibly distended (Fig. 2). Depending on the size of the animal, the inflation may take 5–8 ml of collagenase. After inflation, the pancreas is removed by tearing and cutting away from the attached tissue and ducts. For maximum yield, attention is paid to remove the entire pancreas.

#### Islet isolation protocol

**Fig. 3:**
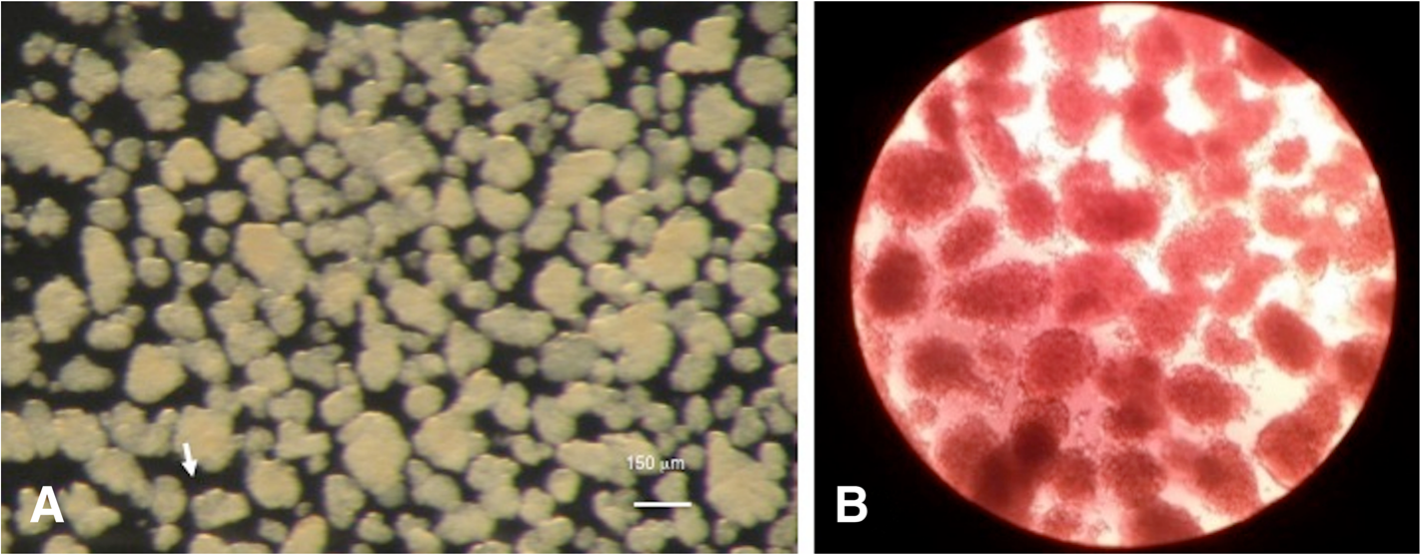
Islets (arrows) seen at the microscope (**a**), and after coloration with dithizone (**b**)

The procedure has been previously described in detail [21]. In brief, the excised pancreas is washed in cold collagenase solution, then placed in a 25 cm^2^ flask and kept on ice until all other pancreata are procured. Usually, no more than two pancreata are placed in one flask. An additional 2–4 ml collagenase is also placed in the flask before incubation. The flask is incubated at 37 °C for 20–25 min without shaking. After incubation the flask is shaken sharply to disrupt the tissue. The digested tissue is placed in a 50 ml conical tube and filled to 50 ml with HBSS-BSA to wash. The tubes are gently centrifuged at 1100 Revolutions Per Minute (RPM) for 1 min to pellet the tissue. The supernatant is removed without disturbing the loosely compacted pellet. It is washed twice more and the supernatant is removed. The tissue is mixed with 25% type 400 Ficoll and a density gradient is constructed by overlaying solutions of 23, 20.5% and finally 11% Ficoll. After centrifugation, collect the interface from each density and wash away the Ficoll with HBSS-BSA solution. Place the collected tissue in a 60 mm non-tissue culture sterile Petri dish. The islets are then handpicked to eliminate any remaining exocrine tissue and immediately counted after isolation by microscopy (Fig. 3). Individual rat islets may range from smaller than 50 to over 400 μm in size; the adult animals used in this study generally had islets in the same proportionate size ranges. On average, this isolation method reliably recovers 150–300 islets from an adult rat (> 25 g body weight). Whole islets, are maintained in complete medium at 37 °C in a humidified atmosphere of 95% air and 5% CO_2_.

#### Islet transplantation in recipient diabetic rats

**Fig. 4:**
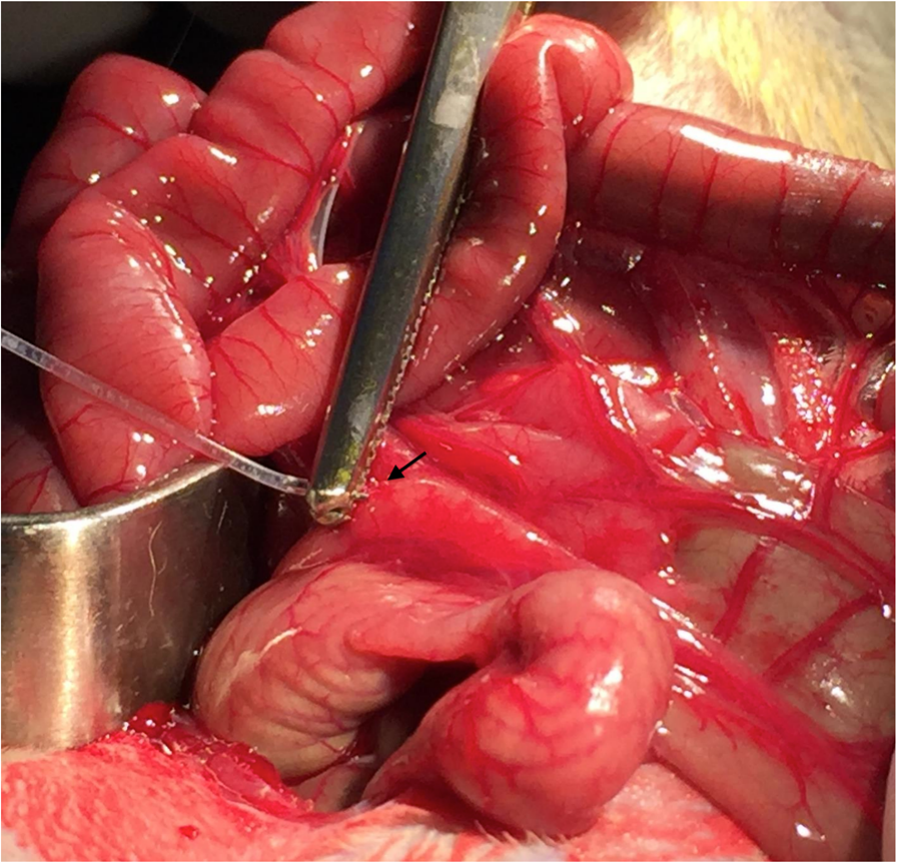
The islets are slowly injected into the mesenteric lymph node by using a cannula (arrow)

The procedure was carried out under sterile conditions. The animals are, first of all, anesthetized with gaseous anesthesia (isofloran/oxygen mixture 2–4% 1–2 L/min) and a laparotomy is performed; a surgical retractor is used to improve the access and visibility of the abdominal cavity. The bowel is pulled out and the mesenteric lymph nodes are located. The islets, previously loaded into a 1.5 ml tube with their proper medium, are slowly injected into the mesenteric lymph node by using a cannula (Fig. 4). To provide some indications of the approximate site of the islet transplant, a 5–0 Prolene® marking stitch is applied. The bowel is repositioned back into the abdominal cavity. The body wall is sutured with 6–0 Nylon. The procedure is completed in about 15 min. The animals are kept warm until recovery from anesthesia.

Necropsy was performed in all transplanted animals. Biopsies of the transplanted and non-transplanted LN were performed for microscopic examination for the presence of insulin-positive cells. Tissues were fixed in 10% buffered formalin, and paraffin sections were stained with hematoxylin and eosin (H&E) insulin and glucagon, using standard procedures. Immunofluorescence studies (TUNEL) for apoptosis were carried out on frozen sections. The pathologist (LP) was blinded to result analysis.

Lymph nodes were treated with 10% formalin for 24–48 h and for immunohistochemical analyses. Each sample was treated with Anti-Insulin Antibodies, (Dako, Glostrup, Denmark); anti-sinaptofisina, clone SY38 (Dako, Glostrup, Denmark); anti-chromogranin A, (Dako, Glostrup, Denmark); anti-CD68, clone PG-M1 (Dako, Glostrup, Denmark); anti-CD68, clone KP1 (Dako, Glostrup, Denmark).

The primary outcome of the study was the return to euglycemia without the need of insulin therapy, in diabetic rats undergoing islet transplantation; and, demonstrating viable islets in recovered lymph nodes.

All the experiments were conducted by the same investigators (LV, RB, SB, MV).

### Statistical analysis

Levels of glycemic were expressed as mean values and comparison of means pre- and post- islet transplantation were estimated by the unpaired two Student’s t-test or Mann-Whitney U test, as appropriate. *p* value < 0.05 was considered as statistically significant. All calculations were performed using SPSS, version 12.0.

## Results

A total of 7 islet transplantations in mesenteric lymph nodes have been performed. Two rats died 24 and 36 h after transplantation due to complications.

Three groups of rats were considered:

- ○ Group A: three diabetic rats;
- ○ Group B: three non-diabetic rats (controls);
- ○ Group C: five diabetic rats receiving islet transplantation.

**Fig. 5:**
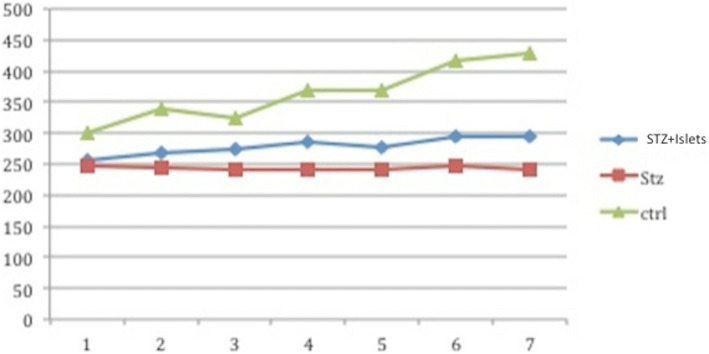
Change in body weight in experimental groups; STZ + islet (transplanted rats); STZ (diabetic rats not transplanted); ctrl (controls)

Five transplanted rats survived until euthanasia through CO_2_ inhalation. No transplanted rat acquired normal glucose blood levels and insulin independence after the transplantation. However, the mean blood levels of glycemia were significantly lower in transplanted rats compared with diabetic rats (470.4 mg/dl vs 605 mg/dl, p 0.04). Interestingly, transplanted rats have a significant weight increase after transplantation compared to diabetic rats (mean value 295 g in transplanted rats vs 245 g in diabetic rats, *p* < 0.05) (Fig. 5), with an overall improvement of social activities and health. There was no significant correlation between the number of transplanted islets and levels of glycemia.

As expected, there was no evidence of islet in mesenteric lymph-nodes in group A and B rats.

**Fig. 6:**
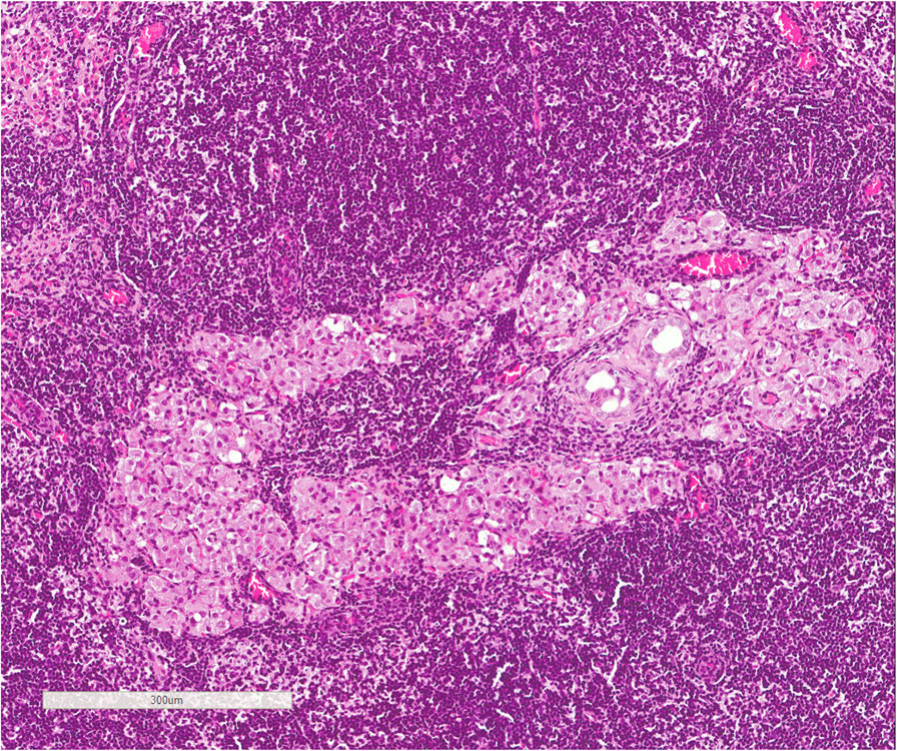
Hematoxylin and eosin section demonstrating the presence of viable islets into the lymph node

**Fig. 7:**
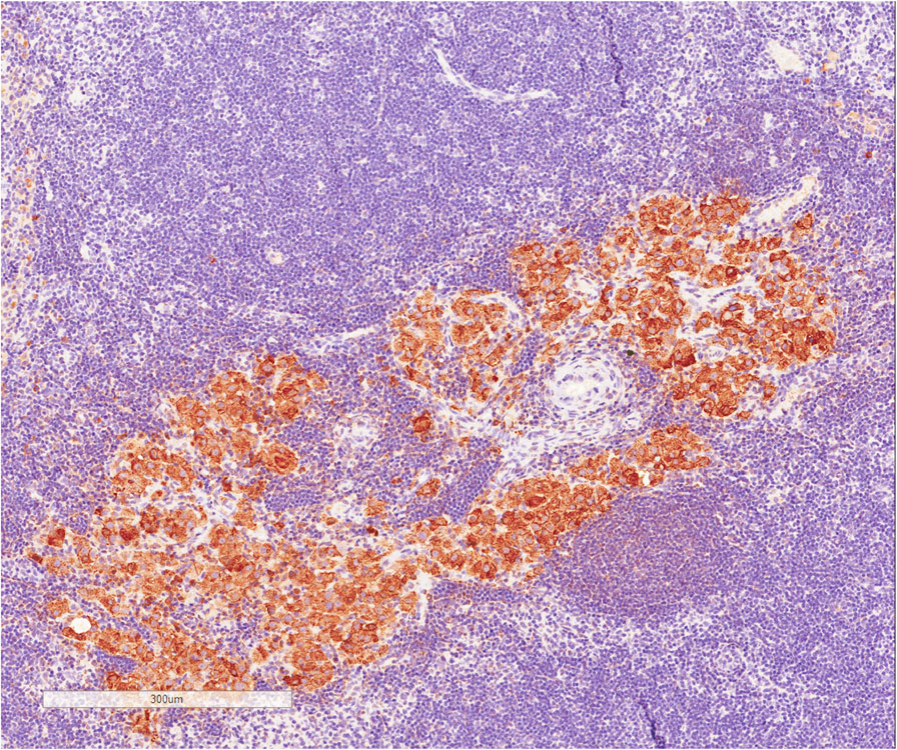
Insulin positive cells in transplanted lymph-node

**Fig. 8:**
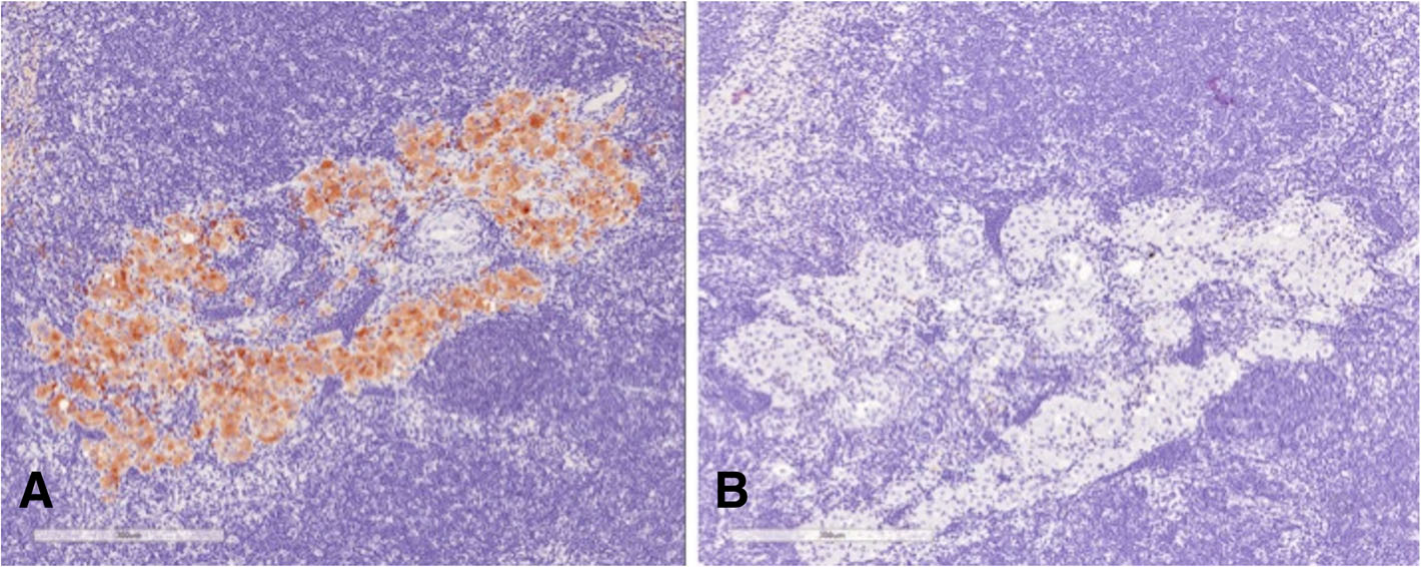
Chromogranin staining examination demonstrated the presence of viable β pancreatic cells (**a**) with no α-cells (**b**)

**Fig. 9:**
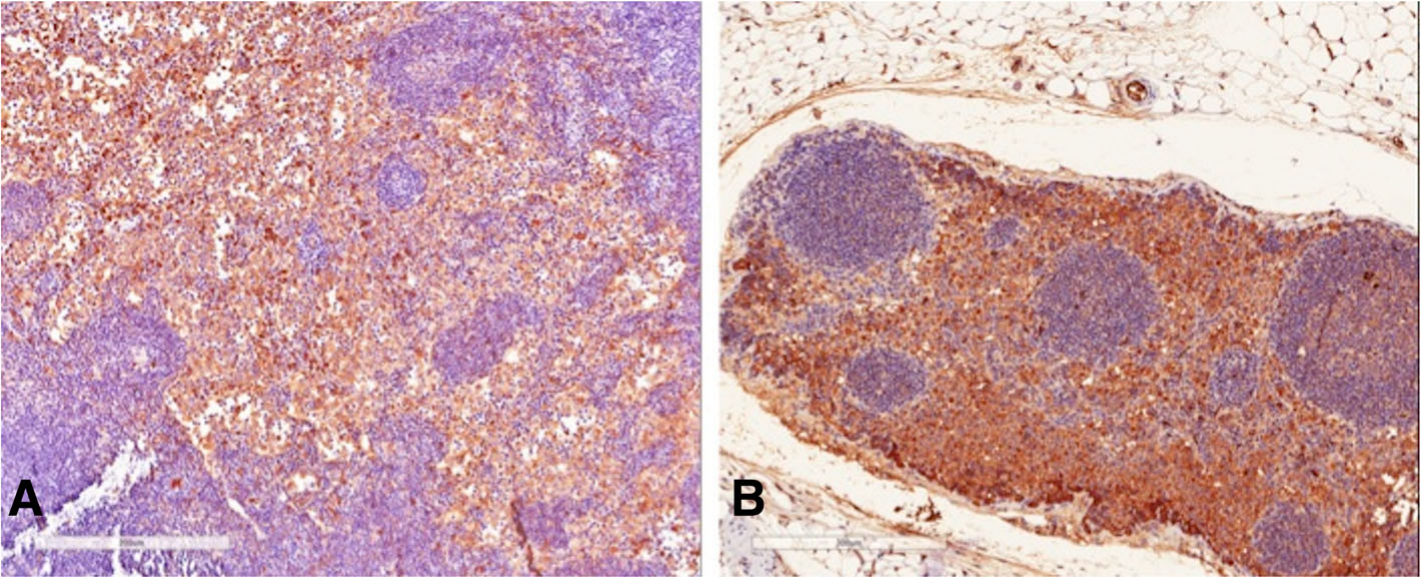
Immunohistochemical analyses demonstrated absence of intact islets/beta cells (**a**), with macrophages elements positive for insulin (**b**)

Immunohistochemical analysis of the 5 mesenteric lymph nodes of transplanted rats demonstrated the presence of living islets in one lymph node (Fig. 6), as confirmed by the presence of insulin-positive cells (Fig. 7) and anti-sinaptofisina, clone SY38 and anti-chromogranin A analyses (Fig. 8). In other transplanted lymph-nodes, there was no evidence of intact islet cells, but only macrophage elements positive for insulin, suggesting the existence of necrotic or apoptotic islets (Fig. 9).

## Discussion

The research for alternative transplant sites plays a fundamental role in the field of pancreatic islet transplantation and, in general, of cellular transplantation. The results reported by several previous studies show the gastric submucosal space is one of the most promising sites that could be clinically used in the near future [14–16]. However, this assumption does not appear to be completely valid for the lymph nodes, whose use in islet transplantation is possible but it may be intended to better characterize the mechanism of allo- and xeno-transplantation of pancreatic islets in animal models.

The present study allowed obtaining more information about some aspects of the islet transplantation already highlighted by previous studies. The intent to prove whether the anatomical location of the lymph nodes affects islet survival and function was inspired by Komori’s work [19], who hypothesized and evaluated the surprising potential of lymph nodes as transplantation sites for multiple tissues. In their work, Komori et al [19],underlined the interesting properties of the lymph nodes: their specific environment protects and enhances survival of lymphocytes, they are close to blood vessels and they also contain fibroblastic reticular cells and other stromal cells that secrete chemokines. They engrafted three distinct healthy cell types, hepatocytes, thymic tissue and pancreatic islets, in different lymph nodes of mice and they observed their ability to promote survival and growth of transplanted cells [19]. Syngeneic mouse hepatocytes marked with green fluorescent protein were injected into the single large jejunal lymph node – it was selected because it is easily accessible and it is the largest in the mouse; one week after injection, the cells were retained mainly in the subcapsular sinus of the lymph node rather than the follicles or germinal centres and, additionally, they formed patches of tissue accompanied by remodelling of blood vessels and growth [19, 22]. Similarly, they injected thymic tissue into the same lymph node of athymic mice and ten months after they observed its long-term engraftment, confirmed by demonstrating that a T-cell-dependent immunity had been acquired by the recipients [19, 22]. Concerning pancreatic islets, the study showed successful islet transplantation in a diabetic syngeneic mouse model, by the infusion of 300 mouse islets mixed with Matrigel into the jejunal lymph node of mice treated with streptozotocin; as well as the hepatocytes, the islets were found in the subcapsular sinus of the lymph node and the expression of C-peptide was detected. Six weeks after transplantation the authors observed the normalization of blood glucose in the recipients, demonstrating the survival of pancreatic islets and the ability of the lymph nodes to sustain long-term normoglycaemia [19, 22]. Then, according to the immunological role of the lymph nodes, they hypothesize that an immune response might interfere with the function of the engrafted cells, so they induced an inflammatory reaction in the intraperitoneal cavity of normoglycaemic recipients with an injection of lipopolysaccharide: they observed a temporary reduction in mouse weight and blood glucose levels, but any increase in glucose blood levels above normal levels was observed, which meant functioning grafts [19, 22]. These data suggested that the lymph nodes did not have negative effects on grafted islets. Their analysis included two other important findings: they observed many endothelial cells expressed by grafted islet around the areas of the engraftment, suggesting that extensive vascular remodelling takes place during the engraftment [19]; additionally, they detected markers of neovascular remodelling in each of the engrafted lymph nodes, suggesting that blood vessels surrounding the lymph nodes also contribute to the neovascularization [19]. These observations allow the establishment of the lymph node as a new potential site for functional cellular transplant, taking account of other properties in addition to those already mentioned: its accessibility and the possibility to monitor cells’ function by its biopsy or even its excision; moreover, in the islet transplantation, another advantage could be the secretion of insulin directly into the portal circulation by using the lymph nodes of the abdominal cavity. The results of the present study, at least in part, confirm the hypothesis by Komori and colleagues [19], considering the following evidences: a) the blood glucose levels in transplanted rats were significantly lower than those in diabetic rats; b) transplanted rats’ weight was increased after islet transplantation; c) transplanted rats’ social activities and health were improved after transplantation; d) living islets were immunohistochemically demonstrated in one mesenteric lymph node. However, although the studies by Komori’s group [19] suggest that the lymph nodes are a suitable site for islet transplantation, it is at the same time possible that the lymph node may represent an adverse and more immunogenic site and that in the absence of immunosuppression allogeneic islets may be rejected very rapidly, and this is probably independent by the site used. This possibility would explain a fifth evidence of the present study: e) why in some transplanted lymph-nodes there was no evidence of liable islet cells, but only macrophage elements positive for insulin, suggesting the islet apoptosis. If this proves to be the case, nevertheless syngeneic islets show prolonged survival and function, we will still progress to studies in cynomolgus monkeys where immunosuppressive therapy will be administered, in contrast to rats which do not tolerate well immunosuppression.

## Conclusions

Our study demonstrated that, although islet engraftment in lymph nodes is possible, islet transplantation in lymph nodes in rats resulted in few improvements of glucose parameters. Nevertheless, this study provided a step forward in the knowledge of the mechanisms of islet engraftment.

## Abbreviations

FBS: Fetal bovine serum
GSMS: Gastric sub-mucosal space
H&E: Hematoxylin and eosin
HBSS-BSA: Hepes plus 0.2% BSA
HBSS-Hepes: Hepes
IBMIR: Blood-mediated inflammatory reaction
LN: Lymph nodes
RPM: Revolutions Per Minute
STZ: Streptozotocin
